# Intensity-modulated radiotherapy reduces gastrointestinal toxicity in pelvic radiation therapy with moderate dose

**DOI:** 10.1371/journal.pone.0183339

**Published:** 2017-08-28

**Authors:** Yoo-Kang Kwak, Sea-Won Lee, Chul Seung Kay, Hee Hyun Park

**Affiliations:** 1 Department of Radiation Oncology, Seoul St. Mary’s Hospital, College of Medicine, The Catholic University of Korea, Seoul, Republic of Korea; 2 Department of Radiation Oncology, Incheon St. Mary’s Hospital, College of Medicine, the Catholic University of Korea, Seoul, Republic of Korea; Taipei Medical University, TAIWAN

## Abstract

This retrospective study was performed to evaluate and compare gastrointestinal (GI) toxicities caused by conventional radiotherapy (cRT) and intensity modulated radiotherapy (IMRT) in 136 cancer patients treated with pelvic radiotherapy (RT) with moderate radiation dose in a single institution. A matched-pair analysis of the two groups was performed; each group included 68 patients. Conventional RT was delivered using the four-field box technique and IMRT was delivered with helical tomotherapy. The median daily dose was 1.8 Gy and the median total dose was 50.4 Gy (range 25.2–56 Gy). Primary end point was GI toxicity during and after RT. Secondary end point was factors that affect toxicity. Patients treated with IMRT had lower incidence of grade ≥ 2 acute GI toxicity compared to the patients treated with cRT (*p* = 0.003). The difference remained significant in multivariate analysis (*p* = 0.01). The incidence of chronic GI toxicity was not statistically different between the two groups, but the cRT group had higher incidence of grade 3 chronic GI toxicity. Based on our results, IMRT can reduce GI toxicity compared to cRT in the treatment of pelvic radiotherapy even with moderate radiation dose and this will enhance patients’ quality of life and treatment compliance.

## Introduction

Indications for radiotherapy are gradually increasing and radiotherapy is applied in various cancer treatments. Particularly, radiotherapy plays a major role in the curative and adjuvant treatment of cancer that develops in the pelvis. For an example, in uterine cervical cancer, treatment outcomes after radiotherapy or surgery are comparable in early staged disease [1] and in cases of locally advanced diseases, chemoradiotherapy is the mainstay of treatment [2–4]. Unfortunately, treating patients with radiotherapy is like a double-edged sword. Although radiotherapy is beneficial to eradicate the cancer cells, damage to the adjacent normal tissues is inevitable. Particularly, when delivering radiation to pelvis, gastrointestinal complications are always of first concern due to its proximity to the target volumes and since the tolerable radiation dose of bowel is lower than the sufficient treatment dose to the cancer cells [5].

Over the past hundred years, radiotherapy techniques and equipment have evolved in terms of conformality, dose escalation, and normal tissue sparing. Intensity modulated radiotherapy (IMRT) has come into the spotlight and is becoming generalized nowadays. IMRT can achieve greater conformity by optimally modulating the intensity of individual beams and more homogeneous dose distribution can be achieved with sharper fall-off of dose at target boundaries thereby sparing adjacent normal tissues, thereby reducing the toxicity. Therefore, we performed this study to compare and evaluate acute and chronic gastrointestinal toxicities caused by pelvic radiotherapy with moderate dose with conventional radiotherapy (cRT) using the four-field box technique and IMRT using helical tomotherapy.

## Materials and methods

The data used in this retrospective study were fully anonymized before access by the researchers and the IRB waived the requirement for informed consent. An approval of our institution’s internal review board (OC14RISI0056) was achieved. Medical records of patients treated with pelvic radiotherapy, from January 2006 to March 2012, were reviewed. Since the aim of this study was to compare the GI toxicity, GI malignancies such as colorectal cancer were excluded. Also, since this study was performed to evaluate and compare the effect of moderate radiation dose on GI toxicity treated with IMRT and cRT, prostate cancer was excluded, because prostate cancer is treated to more than 70 Gy. Benefits of IMRT compared to cRT in high radiation dose is already well-known. Therefore, patients diagnosed with uterine cervical cancer, endometrial cancer, and bladder cancer were enrolled. Of 179 patients identified, 68 patients were treated with IMRT and 111 patients were treated with cRT. We performed a matched-pair analysis of the two groups, and the 43 patients in cRT group that were unfit for analysis were excluded. Therefore, 68 patients in each group were assessed.

### Assessment

Chart review focused on the patient’s age, ECOG performance status, tumor histologic types, treatment prior to radiotherapy, radiotherapy modality, chemotherapy, and cancer stage. Radiologic examinations such as computed tomography (CT), magnetic resonance imaging (MRI), or PET-CT were also reviewed to determine the initial stage and evaluate responses after treatment. The American Joint Committee of Cancer (AJCC) 7^th^ edition was used for staging.

### Radiotherapy

Simulation was done in supine position with both arms up together and whole body Vac-Lok^TM^ (CIVCO Medical Solutions, Coralville, Iowa) was used for immobilization. Intravenous contrast-enhanced CT images taken by Light Speed^R^ RT 16 CT scanner (GE Healthcare, Waukesha, WI) were achieved with 3mm- or 5mm-slice intervals, from cranial border of the L2 vertebral body to 5cm below the ischial tuberosity, at least one week before the beginning of radiotherapy.

IMRT was delivered using helical tomotherapy (Accuray Inc., Sunnyvale, CA). Gross primary tumor and all visible lymph nodes were delineated slice by slice. Regional lymphatics included the common, external, and internal iliac and obturator nodes. As reference guidelines on contouring pelvic lymph nodes developed, relatively uniform contours of regional lymph nodes were achieved [6,7]. Organs-at-risk was small bowel, delineated from L4 level and below. For IMRT, the gross tumor volume (GTV) was defined as visible tumor, including the primary lesion and enlarged lymph nodes; the clinical target volume (CTV) included the involved whole organ with 1cm margin added around the gross tumor and regional lymph nodes. In reference to previously reported data on internal pelvic organ motions [8–10] and with a concern for errors during the set-up, additional margins of 0.5cm surrounding the CTV was defined as the planning target volume (PTV). Treatment planning was done using Tomotherapy Planning System (Accuray Inc., Sunnyvale, CA). The median dose was 50.4 Gy for PTV and target planning constraints were as follows: (1) more than 95% of PTV receives at least 95% of the prescription dose; (2) less than 1% of PTV receives more than 107% of the prescription dose; and (3) the maximum dose was below 115% of the prescription dose. As a result, the average PTV coverage was 96%. There were no hot spots in the small bowel.

Conventional radiotherapy was delivered using the four-field box technique with ClinacIx linear accelerator (Varian Medical Systems, Palo Alto, CA) in 6 to 15 MV. For cRT, Eclipse^TM^ver 8.9 Treatment Planning System (Varian Medical Systems, Palo Alto, CA) was used. The anterior/posterior (AP/PA) field borders were as follows: L5/S1 junction for superior border; lower border of the ischial tuberosity for inferior border; and 1.5cm lateral to the pelvic rim for lateral borders. In the lateral fields, superior and inferior borders were the same as the AP/PA field borders, while anterior border was the front of the pubic symphysis, and posterior border was the junction of S2/S3, or occasionally included the whole sacrum, depending on the extent of the primary tumor.

After whole pelvis external radiotherapy, patients received boost radiotherapy using intracavitary radiotherapy (ICR), IMRT, or 3D-conformal RT, if clinically indicated.

### Follow-up

Since the beginning of the treatment, patients were interviewed weekly upon clinical examination during the period of radiotherapy. Patients were re-evaluated two weeks after completing the treatment, then monthly for 3 months, and then at 2- or 3-months intervals. Patients were assessed on acute toxicities throughout the course of treatment and up to 3 months after the treatment. Chronic toxicities were defined as toxicities developed after 3 months of RT completion. Toxicities were graded according to the National Cancer Institute Common Terminology Criteria for Adverse Events (CTCAE) v4.0.

### Statistics

Primary end point of this study was the difference in the incidence of GI toxicities and survival between the two RT modalities. Secondary end point was the factors that affect treatment toxicity. To eliminate the disparity between the two groups, matched-pair analysis was performed. Chi-square or Fisher’s exact test was used to compare the incidences of categorical variables and Student’s t-test was used for continuous variables. For univariate and multivariate analyses, logistic regression analysis was used. When interpreting the results, *p-*values less than 0.05 were considered statistically significant. All data were computed and analyzed with SPSS for Windows, version 12.0 (SPSS Inc., Chicago, IL, USA).

## Results

### Patients

Patients’ baseline characteristics are shown in Table 1. The proportion of uterine cervical cancer was major, because it is the most common pelvic malignancy and definitive or adjuvant aimed radiotherapy is very beneficial in various stages of the disease. Most of the patients had ECOG performance of 0 or 1 and were in early stage. Around 80% of patients received surgery prior to radiotherapy and about half the patients was treated with concurrent chemotherapy. More than 95% of the patients received radiotherapy to the routine whole pelvis. Radiotherapy to the whole pelvis was delivered at a median total dose of 50.4 Gy (range 25.2–56 Gy), once daily, 5 times per week, 1.8 Gy per fraction (range 1.8–2.0 Gy). The median treatment time was 42 days for the cRT group and 40 days for the IMRT group. In the IMRT arm, boost RT was delivered in 19 patients (27.9%): 15 patients received ICR at a median dose of 20 Gy in 5 fractions and 4 patients received IMRT boost. In the cRT arm, 20 patients (29.4%) received boost radiotherapy. Of them, 14 patients received intracavitary radiotherapy (ICR) at a median dose of 30 Gy in 6 fractions, 3 patients underwent IMRT boost, and 3 patients had 3-dimentional conformal radiotherapy. ICR was mainly used in gynecological cancer, especially in cervix cancer. Since ICR is a technique that places radioactive material directly to body cavity, the indication is limited to organs that are easily approachable, such as vagina. One of the advantages of ICR is the direct radiation delivery to the cancerous lesions with short-range radiation, which can minimize radiation influence to other organs like small bowel. The difference of patients’ characteristics between the IMRT and cRT group was not found.

**Table 1:** Patient characteristics.

	IMRT (n = 68)	cRT (n = 68)	*p-*value
Characteristics	No. (%)	No. (%)	
Age	Median 55 years		0.49
≤ 55	38 (55.9)	33 (48.5)	
> 55	30 (44.1)	35 (51.5)	
Cancer type			0.19
Uterine cervix	48 (70.6)	57 (83.8)	
Endometrial	18 (26.5)	9 (13.2)	
Bladder	2 (2.9)	2 (2.9)	
ECOG performance			0.77
0	21 (30.9)	18 (26.5)	
1	41 (60.3)	43 (63.2)	
2	6 (8.8)	7 (10.3)	
Stage			0.74
I-II	62 (91.2)	59 (86.7)	
III-IV	6 (8.8)	9 (13.2)	
Histology			0.43
Sqaumous ca.	32 (47.1)	40 (58.8)	
Adenoca.	25 (36.8)	19 (27.9)	
Others	11 (16.2)	9 (13.2)	
Pre-RT surgery			0.51
No	11 (16.2)	15 (22.1)	
Yes	57 (83.8)	53 (77.9)	
Pre-RT chemotherapy			0.83
No	54 (79.4)	56 (82.4)	
Yes	14 (20.6)	12 (17.6)	
Concurrent Chemotherapy			0.23
No (RT alone)	36 (52.9)	28 (41.2)	
Yes	32 (47.1)	40 (58.8)	
RT dose			0.24
< 50.4 Gy	18 (26.5)	11 (16.2)	
50.4 Gy	42 (61.8)	44 (64.7)	
> 50.4 Gy	8 (11.8)	13 (19.1)	
RT field			0.93
Routine pelvis	66 (98.5)	67 (98.5)	
Extended field	1 (1.5)	1 (1.5)	
RT boost			0.85
No	49 (72.1)	48 (70.6)	
Yes	19 (27.9)	20 (29.4)	

### Toxicities

Chi square test was used to compare the occurrence of acute and chronic toxicities between the IMRT and cRT groups. Since medical intervention is necessary with grade 2 or greater toxicity and the need of medication can influence on patients’ quality of life, we assessed on acute toxicity of grade 2 and above. Acute and chronic toxicity rate of grade 2 and above were 52.9% and 11.0%, respectively.

Univariate and multivariate analyses to determine the parameters that affect acute and chronic GI toxicity were performed (Table 2). Regarding acute toxicity (grade ≥ 2), cancer type, cancer histology, concurrent chemotherapy, radiotherapy dose, and radiotherapy modality showed statistical significance in univariate analysis. In multivariate analysis, cancer type (*p* = 0.001) and radiotherapy modality (*p* = 0.01) showed statistical significance in the development of acute GI toxicity. Concerning chronic toxicity, cancer type had statistical significance and histology showed marginal significance in univariate analysis, but these did not lead to significant results in multivariate analysis.

**Table 2:** Factors that affect toxicity in pelvis radiotherapy.

	Acute toxicity (grade ≥ 2)			Chronic toxicity		
Factors	Univariate (*p*)	HR (95% CI)	Multivariate (*p*)	Univariate (*p*)	HR (95% CI)	Multivariate (*p*)
Age, year (≤ 55 vs > 55)	0.09			0.53		
Cancer type	**0.00**		**0.001**	**0.004**		0.27
Uterine cervical		**1.00 (referent)**			1.00 (referent)	
Endometrial		**0.02 (0.002–0.23)**			0.24 (0.04–1.36)	
Bladder		**0.00**			0.00	
ECOG (0–1 vs 2–3)	0.70			0.96		
Stage (I-II vs III-IV)	0.32			0.12		
Histology	**0.003**		0.19	**0.08**	0.76	
Adenocarcinoma		1.00 (referent)			1.00 (referent)	
Squamous cell carcinoma		0.41 (0.13–1.30)			0.64 (1.93–2.10)	
Pre-RT surgery	0.07	1.48 (0.49–4.52)	0.49	0.45		
Pre-RT chemotherapy	0.16			0.66		
Concurrent chemotherapy	**0.00**	**0.02 (0.95–5.15)**	**0.06**	0.65		
Radiotherapy dose	**0.03**		0.52	0.70		
< 50.4 Gy		1.00 (referent)				
50.4 Gy		0.45 (0.11–1.80)				
> 50.4 Gy		0.49 (0.09–2.79)				
Radiotherapy field	0.86			0.73		
Boost radiotherapy	0.89			0.72		
Radiotherapy modality	**0.003**		**0.01**	0.12		0.22
IMRT		**1.00 (referent)**			1.00 (referent)	
cRT		**3.39 (1.20–6.43)**			1.70 (0.72–3.98)	

**Fig 1:**
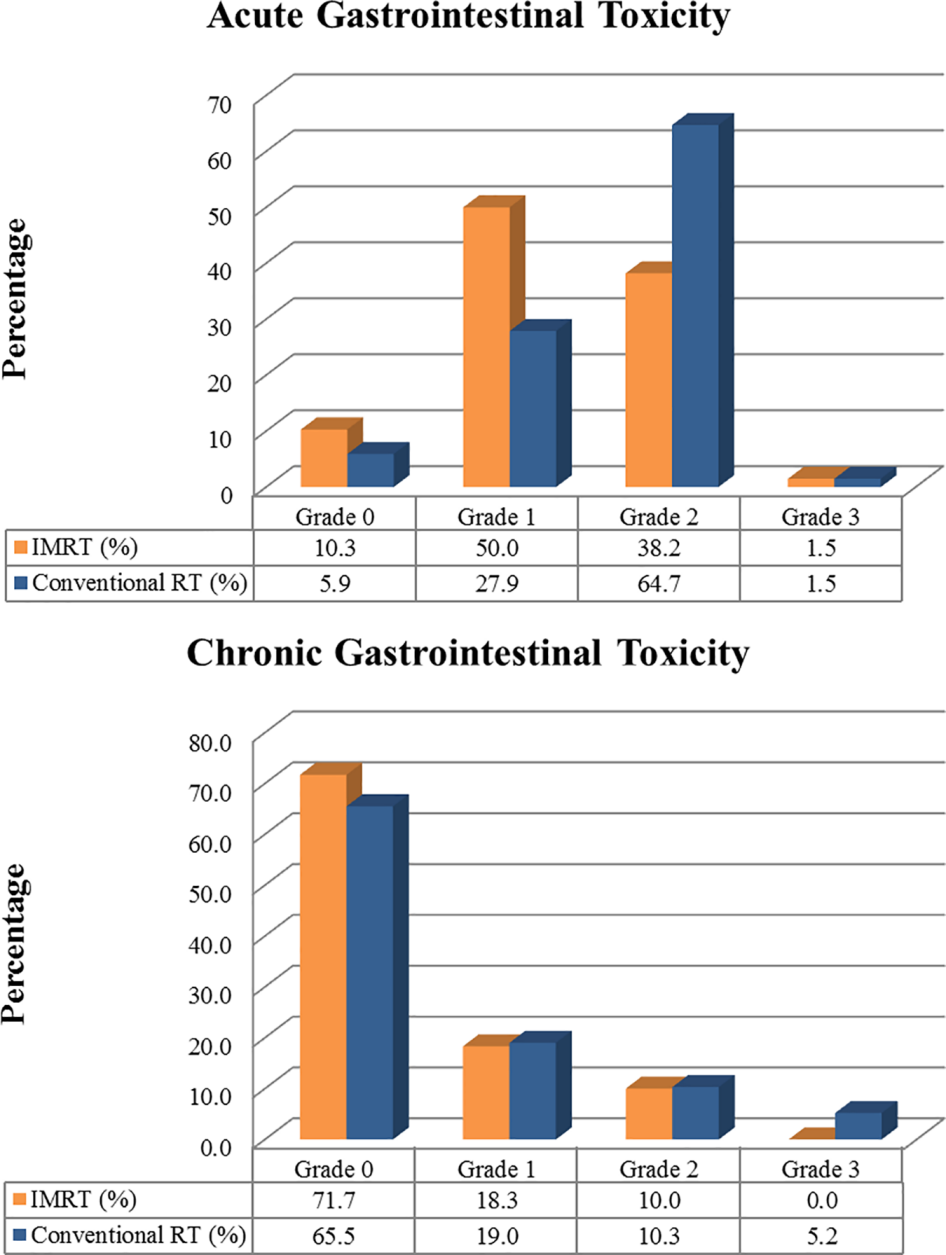
Incidence of GI toxicity according to radiotherapy technique. (a) Incidence of acute GI toxicity (b) Incidence of chronic GI toxicity.

The crude incidence of acute GI toxicity is shown in Fig 1A and the incidence of grade 2 acute toxicity in cRT group was almost as twice as that of IMRT group. Grade ≥ 2 of acute gastrointestinal (GI) toxicity occurred less in the IMRT group with statistical significance with *p-*value of 0.003. Odds ratio for cRT group to develop grade ≥ 2 GI toxicity was 3.39, compared to the IMRT group with 95% confidence interval of 1.20–6.43. Chronic toxicity was evaluated in 60 patients in the IMRT group and 58 patients in the cRT group. There were no statistical differences in terms of chronic toxicity between the two groups. The crude incidence of chronic toxicity is depicted in Fig 1B. In both groups, more than two thirds of patients did not develop chronic toxicity. Totally, 37 patients (26.8%) complained of GI symptoms after 3 months of RT completion; 17 patients in the IMRT group, 20 patients in the cRT group (*p-*value = 0.15). Three patients (5.2%) in the cRT group suffered grade 3 chronic toxicity; one with rectovaginal fistula, and the others with enteritis. There were no grade 3 chronic toxicity in the IMRT group, but the difference between the two groups was not statistically significant (*p-*value = 0.12) due to small number of events. There were no grade 4 chronic GI toxicities or treatment related deaths in both IMRT and cRT group.

### Dosimetric comparison of IMRT and cRT

In the IMRT group, mean small bowel dose was 24.1 Gy and mean bowel volume above 35 Gy was 20.6%, above 45 Gy was 8%, and above 50 Gy was 1.5%. Mean bowel dose in the cRT group was 28.6 Gy. In the cRT group, mean bowel volume above 35 Gy was 40.6%, above 45 Gy was 30.3%, and above 50 Gy was 9%. Statistical analysis of dosimetric comparison between the two groups showed significance in only small bowel dose above 50 Gy with a *p-*value of 0.04.

## Discussion

**Fig 2:**
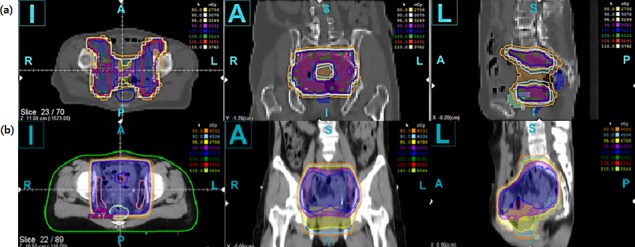
**CT slice of IMRT (a) and cRT (b) radiotherapy plans with isodose lines (80%, orange; 95%, yellow; 100%, blue; 103%, green)**. Note that small bowel and rectum in IMRT plan are spared. (a) IMRT, Intensity-modulated Radiotherapy. (b) cRT, Conventional Radiotherapy.

Radiotherapy plays an essential role in radical and adjuvant treatment of cancer that develops in the pelvic cavity, which makes it a crucial treatment modality. However, unfortunately, toxicities caused by radiotherapy still remain to be solved. IMRT has become more prevalent as the understanding of radiation and radiotherapy techniques improved. IMRT can deliver highly conformal radiation to the targets and simultaneously constrain the radiation dose to the organs-at-risk, which enables to spare normal organs while delivering adequate radiation to the targets. Therefore, with IMRT, sparing the small bowel is obtainable while sufficiently covering the targets. In conventional RT with four-field box technique, small bowel within the radiation field is homogeneously irradiated with the targets. Several dosimetric studies have already proven the hypothesis that IMRT can decrease radiation to the organs-at-risk [11–15]. In a dosimetric analysis carried out by Roeske et al, the volume of small bowel irradiated above 45 Gy was significantly associated with increased GI toxicity(*p =* 0.009) [16]. Dosimetric analyses in our study also showed superiority in IMRT plans. An example of both plans is depicted in Fig 2A and 2B. In our study, the small bowel volume above 45 Gy and 50 Gy in the cRT group was higher than that of IMRT group. In the same context, this study demonstrated significantly lower rate of grade ≥ 2 GI toxicities in the IMRT group than the cRT group with statistical significance (*p =* 0.003; OR = 2.97; 95% CI, 1.48–5.98) and the significance was retained after controlling other factors (*p =* 0.01; OR = 3.39; 95% CI, 1.20–6.43). Although the difference was not statistically significant, the crude incidence of chronic GI toxicity was higher in the cRT group than the IMRT group.

Consistent with these dosimetric data, reduced toxicities in the practice of treating cancer in the pelvic cavity were anticipated with the use of IMRT. Some retrospective studies compared the incidence of toxicities in IMRT and cRT, and presented positive results of IMRT [17–20]. In a prospective study directed by Gandhi, 44 patients were randomized to cRT or IMRT for 50.4 Gy of radiotherapy in 28 fractions. They reported on fewer grade ≥ 2 acute GI toxicities (*p* = 0.034) and lower incidence of chronic GI toxicities (*p* = 0.011) in the IMRT arm with statistical significance [21]. Additionally, there are many retrospective studies that have proven the beneficial effect of IMRT in reducing toxicity [18,22,23]. However, the mentioned studies included only a small number of patients and their cohorts were heterogeneous. In this study, we performed a matched-pair analysis to diminish the discrepancies between the comparing groups and the number of enrolled patients was not small. Nevertheless our results showed reduced incidence of grade 2 GI toxicity in IMRT with statistical significance.

Toxicity evaluation is an unsolved problem since it has to reflect patients’ subjective symptoms while sustaining unbiased assessment. There are several challenges that need to be solved. First, patients’ symptoms are not always related to signs related to RT changes. Yeoh et al investigated on anorectal dysfunction after radiotherapy [24–26]. In his prospective studies, patients underwent evaluation of anal function using manometric techniques, morphology by ultrasonography, and compliance using rectal balloon during and after radiotherapy. Also, Late Effects in Normal Tissues Subjective, Objective, Management and Analytic scales (LENT-SOMA) questionnaire was given to patients to fill up. In a systematic review, he concluded that objective changes are not always correlated with patient’s subjective symptoms [27]. In our study, objective examinations such as manometry or ultrasonography were not routinely performed during or after radiotherapy because the tests are not covered by national insurance, so it is not cost-effective. As the prospective studies attest, objective changes do not always represent symptoms. Our study focused on patient reported toxicity. In our institution, it is our practice to interview the patients with specific and systematic questions on the symptoms that can be induced by radiotherapy during follow ups. The fact that these recordings were not filled up by patients is a limitation since discrepancies in descriptions on adverse events between clinicians and patients exist. This is another obstacle in toxicity assessment. On this problem, Davidson et al conducted systematic prospective studies in gynecological cancer patients after radiotherapy using questionnaires for a prolonged period [28–30]. Recordings by clinicians were underrated compared to patient’s complaints in most of the cases. Moreover, standardized criteria on toxicity evaluation are not yet adequate. For example, RTOG and CTCAE scales lack some gastrointestinal symptoms such as defecation urgency and fecal incontinence, which can induce an underestimated evaluation. This problem of underestimation is also discussed in previously reported studies [31,32]. Lastly, the pathophysiology of toxicity caused by radiation is not verified.

Even with the limitations on evaluation of toxicity, this study was performed to find out whether advanced radiotherapy technique can influence on the incidence of GI toxicity in pelvic RT. The results of our study proved reduced GI toxicity with IMRT with statistical significance compared to cRT.

## Conclusion

This study was performed to compare the acute and chronic GI toxicities in 136 patients who were treated with IMRT or cRT to the pelvis with moderate radiation dose. The results showed superiority of IMRT over cRT. With reduced toxicity, IMRT will enhance patients’ quality of life and treatment compliance. Our results suggest that with IMRT, selective dose escalation in radiotherapy to the pelvis will be practicable and give the chance of better clinical outcomes. Beforehand, for a more methodical evaluation on toxicity after pelvic radiotherapy, we look forward to investigate a well-planned prospective study with prolonged and thorough follow-up.
